# PS-18 - CO-DEVELOPING MESSAGES TO SUPPORT WINTER VACCINATION FOR PEOPLE EXPERIENCING HOMELESSNESS

**DOI:** 10.1093/pubmed/fdag046.065

**Published:** 2026-07-09

**Authors:** Ms Lianne Straus, Ms Rachel Brennan, Ms Kanwal Faheem, Dr Lois Murray

**Affiliations:** UK Health Security Agency; UK Health Security Agency; UK Health Security Agency; UK Health Security Agency

## Abstract

**Background:**

Insights from people with lived experience of homelessness highlight the need for tailored, co-produced communication to support vaccination uptake. Additionally, given high rates of chronic illnesses, the Joint Committee on Vaccination and Immunisation (JCVI) has recommended an influenza and pneumonia vaccination programme for people rough sleeping or in hostels.

**Objectives:**

This project aimed to co-develop messaging to improve vaccination uptake among people experiencing homelessness, with a focus on influenza and pneumonia.

**Methods:**

Groundswell used a peer-led approach to run four focus groups with 35 people with lived experience of homelessness. Key themes and communication principles were identified. A peer-led group developed draft messages, refined through consultation and reviewed by UKHSA to ensure alignment with public health advice.

**Results:**

Final messages addressed prevention, costs, side effects, protecting others, fear, trust, support, and choice. Principles for communication include: short, clear information; dedicated leaflets; content on what vaccines are and why they matter; varied formats; and trusted delivery routes such as day centres, hostels, peers, and integrated health services.

**Conclusions:**

Co-production with people with lived experience builds trust, accessibility, and engagement. These messages will inform future UKHSA communication materials to support vaccination uptake among people experiencing homelessness. Effective co-production requires time, resources, and commitment across partners. Recognising organisational cultures and power imbalances is essential to fully value and utilise lived experience in shaping access to health protection interventions such as vaccination.

